# Examining reward-seeking, negative self-beliefs and over-general autobiographical memory as mechanisms of change in classroom prevention programs for adolescent depression

**DOI:** 10.1016/j.jad.2015.07.019

**Published:** 2015-11-01

**Authors:** Frances Rice, Adhip Rawal, Lucy Riglin, Gemma Lewis, Glyn Lewis, Sandra Dunsmuir

**Affiliations:** a Department of Clinical, Educational and Health Psychology, University College London, United Kingdom; b Division of Psychiatry, University College London, United Kingdom

**Keywords:** Reward, Autobiographical memory, Negative beliefs, Prevention, Depression, Adolescence

## Abstract

**Background:**

Effective methods to prevent adolescent depressive symptoms could reduce suffering and burden across the lifespan. However, psychological interventions delivered to adolescents show efficacy only in symptomatic or high-risk youth. Targeting causal risk factors and assessing mechanistic change can help devise efficacious universal or classroom based prevention programs.

**Methods:**

A non-randomized longitudinal design was used to compare three classroom-based prevention programs for adolescent depression (Behavioral Activation with Reward Processing, “Thinking about Reward in Young People” (TRY); Cognitive Behavioral Therapy (CBT) and Mindfulness Based Cognitive Therapy (MBCT)), and determine cognitive mechanisms of change in these programs. Cognitive mechanisms examined were reward-seeking, negative self-beliefs (assessed with behavioral tasks) and over-general autobiographical memory. 256 healthy adolescents aged 13–14 participated with 236 (92%) and 227 (89%) completing the pre- and post-assessments.

**Results:**

TRY was the only intervention associated with a reduction in depressive symptoms at follow-up. Reward-seeking increased following TRY. In the other programs there were non-significant changes in cognitive mechanisms, with more reflective negative self-beliefs in CBT and fewer over-general autobiographical memories in MBCT In the TRY program, which focused on increasing sensitivity to rewarding activities, reward seeking increased and this was associated with decreased depressive symptoms.

**Limitations:**

Due to the infeasibility of a cluster randomized controlled trial, a non-randomized design was used.

**Conclusions:**

Increased reward-seeking was associated with decreased depressive symptoms and may be a mechanism of depressive symptom change in the intervention with a focus on enhancing sensitivity and awareness of reward. This study provides preliminary evidence to suggest that incorporating activities to enhance reward sensitivity may be fruitful in randomized controlled trials of universal prevention programs for depression.

## Highlights

• We compared three classroom interventions (TRY, CBT, MCBT) and control.
• We tested if the interventions changed three cognitive risk factors.
• Reward-seeking increased after TRY and was related to a reduction in depression.
• TRY was superior to the other interventions in terms of reducing depressive symptoms.
• Incorporating reward training into universal prevention programs may be useful.

## 1 Introduction

Depression is one of the leading causes of burden and disability in the world (Ustun et al., 2004). The prevalence increases markedly during adolescence and adolescent depression is associated with serious social and educational impairments, high relapse rates and mental health problems in adult life (Angold et al., 1999; Dunn and Goodyer, 2006; Fergusson et al., 2005; Weissman et al., 1999). Despite this, adolescent depression is under-recognized, treatment effect sizes are modest and most affected individuals do not receive any intervention (Thapar et al., 2012). Effective programs that prevent adolescent onset depression are therefore needed.

Selective and indicated preventive interventions are delivered to sub-clinically symptomatic populations (Garber et al., 2009; Horowitz and Garber, 2006; Merry et al., 2004a; Stice et al., 2009) whilst universal prevention programs are delivered to all members of a group regardless of symptoms. Each approach has advantages and disadvantages but a universal approach, which is the one used in the present study, avoids the need for expensive and imperfect screening procedures and reaches large numbers of individuals including those most vulnerable (Rose, 1992). To date, the majority of prevention programs have used similar approaches to those used in psychological treatments and focus on altering dysfunctional styles of thinking and behavior by challenging negative thoughts (i.e. cognitive therapy based approaches). Although early universal prevention programs using cognitive therapy showed promise (Merry et al., 2004b; Shochet et al., 2001) large scale trials do not support this (Stallard et al., 2012). Different approaches to intervention may be needed for promoting protection against symptoms in unselected groups where individuals may not identify with the symptom-based approaches used to date.

An alternative approach involves aiming to target and change cognitive mechanisms thought to increase the risk of depression because targeting and changing causal factors will alter an outcome (Giesen et al., 2007; Rutter, 2007; Toth et al., 2013). Thus, designing interventions that target potentially causal risk factors and measuring whether interventions do indeed change them is one way of devising efficacious programs (Flay et al., 2005; Kraemer et al., 2002). A need to actively compare different interventions as well as to identify the mechanisms of change has been identified as an important way to expedite innovation and enhance efficacy (Hollon et al., 2002; Merry and Stasiak, 2012).

We selected three cognitive mechanisms for which there was evidence they may be causally involved in adolescent depression (i.e. exist prior to and longitudinally predict the onset of depressive symptomatology). These were 1) reward-processing (Forbes et al., 2007; Rawal et al., 2013b) 2) negative self-beliefs (Abela et al., 2011; Rawal et al., 2013a) and 3) over-general autobiographical memory (Abela et al., 2011; Hipwell et al., 2011; Rawal and Rice, 2012a). We examined whether these were changed through three classroom-based prevention programs which we hypothesized would differentially change these cognitive mechanisms due to the different content and focus of programs. It should be highlighted that some measures of cognition will be more tightly linked to measures of depression due to measurement issues. For instance, greater correlations with depression (which relies on self-reported symptoms) are expected where participants report on their cognitive distortions or biases compared to measures derived from performance on cognitive tasks. It can be argued that performance-based measures provide more objective measures than self-report measures because they allow for the measurement of cognitive biases that may not be open to introspection (Harmer et al., 2009; Rawal et al., 2013a). The use of performance-based cognitive indicators also lessens the likelihood that associations with depression are due to shared method variance which is possible when the same informant rates a risk factor (e.g. cognitive bias) and an outcome (e.g. depressive symptoms) (Rutter et al., 2001). For this reason, we focused on performance-based measures of cognition. We first describe evidence that the three cognitive mechanisms may be causally involved in adolescent depression. We then outline our hypotheses related to cognitive change in the three programs.

Depressed adults and adolescents are insensitive to reward (Eshel and Roiser, 2010; Naranjo et al., 2001). This hypo-sensitivity to reward has been shown to exist prior to and increase risk for later adolescent depression (Forbes et al., 2007; Rawal et al., 2013b). Behavioral activation, which encourages active engagement in interesting and pleasurable activities may increase reward sensitivity (Dichter et al., 2009). Depressed adults and adolescents also show a number of distortions of thoughts and memory that negatively bias the perception and experience of events. Prospective longitudinal studies show that these thought distortions may play a causal role in the onset and maintenance of depression (Beck, 1976; Jacobs et al., 2008; Rood et al., 2009; Watkins, 2008; Williams et al., 2007). Moreover, associations with depression are stronger when endorsements of negative self-beliefs are rapid (and more implicit or automatic) compared to when they are slower (and more reflective) (Sheppard and Teasdale, 2004). Cognitive Behavioral Therapy (CBT) focuses on altering dysfunctional styles of thinking and behavior through challenging such negative thoughts and beliefs. Depression is also associated with difficulties in retrieving specific details of the personal past and this phenomenon of over-general autobiographical memory (OGM) predicts later depressive symptomatology in adolescents (Rawal and Rice, 2012b) and prognosis in depressed adults (Brittlebank et al., 1993; Williams et al., 2007). Mindfulness Based Cognitive Therapy (MBCT) focuses on training attention, acceptance and tolerance of emotional states (Kuyken et al., 2013; Stice et al., 2009) and has been found to reduce OGM in adults (Williams et al., 2000). Accurate knowledge of autobiographical events is important for social problem-solving, the ability to imagine future events, and the regulation of emotional material (Williams et al., 2007).

We compared three types of intervention for which there is evidence they may prevent adolescent depression (Horowitz and Garber, 2006; Kuyken et al., 2013; Merry et al., 2004a; Stice et al., 2009) (Thinking about Reward in Young People [TRY], CBT and Mindfulness-Based Cognitive Therapy [MBCT]. TRY incorporated CBT and behavioral activation and focused on enhancing reward-processing. We predicted that the different interventions would alter different cognitive mechanisms. We asked: 1) Are three potentially causal cognitive risk factors for adolescent depression (reward-processing, negative self-beliefs and over-general autobiographical memory) changed following participation in each intervention? 2) Is change in the cognitive risk factors associated with change in depressive symptomatology? We made the following hypotheses: 1) reward-sensitivity would increase in TRY due to the explicit focus of the program on increasing reward-sensitivity. 2) Negative self-beliefs would reduce and become more reflective in CBT as the program encouraged and supported young people in identifying and evaluating negative thoughts and cognitive distortions. 3) OGM would reduce in MBCT due to the focus on increasing present moment awareness in a non-judgmental way which would encourage participants to encode and retrieve events in more specific ways (Williams et al., 2000).

## 2 Methods

A non-randomized longitudinal design with three intervention conditions (TRY, CBT, MBCT) and one comparison condition was used. 256 adolescents aged 13–14 years attending three schools in the South East of England, UK, participated. Participants were allocated to groups according to therapist availability and school timetabling. Each intervention was delivered to two separate classes in one of the three schools. Comparison participants came from all three schools with two schools including one class and one school including two classes. A non-randomized design was used because a randomized controlled trial (RCT) would necessitate clustering within classes or schools and would therefore involve an extremely large sample. Given that this was a preliminary study to investigate cognitive mechanisms and the program content and materials were developed for this study, a full cluster RCT would have been premature and unfeasible. The manualized interventions involved 8 weekly sessions delivered by Educational Psychologists during the school day. Sessions took place during personal health and social education (PHSE) lessons and lasted 50 min. The TRY program aimed to enhance reward-processing through activities such as illustrating the use of rewarding experiences to lift mood and evaluating potential risk and rewards involved in day-to-day decision making. The CBT program aimed to change negative thinking styles by, for example, encouraging evaluation of thoughts and promoting positive coping styles and problem solving. The MBCT program promoted awareness and acceptance of current thoughts and feelings and aimed to develop regulation of attention and mood through guided meditation and consciously paying attention to breathing. All programs and activities were developed for the purpose of this study and were fully manualized. Further details about program development and interventions are included in Appendix 1. Therapists participated in individual and group supervision on a weekly basis, and checks on the fidelity of the intervention implementation for each approach were conducted throughout the course of intervention (Appendix 1). Fidelity checks showed that manual adherence was high for all interventions (TRY; 85%); (CBT; 88%) and (MBCT; 73%) and did not differ between interventions (*F*=.713, *p*=.519). Observer ratings of adolescent and facilitator engagement were also generally good (TRY 73% adolescent, 79% facilitator: CBT 73% adolescent, 85% facilitator: MBCT 61% adolescent, 78% facilitator) and did not differ between interventions (*F*=.576, *p*=.584 adolescent engagement: *F*=.315, *p*=.738 facilitator engagement). Usual school provision following the PHSE curriculum served as the comparison condition. All conditions included psycho-education about depression (in verbal and/or written form). Psycho-education included describing the symptoms, prevalence and causes of depression as well as guidance on helpful and unhelpful behaviors and how to seek help.

### 2.1 Ethics and consent

The study protocol was reviewed and approved by the university ethics committee. Parents were given the opportunity to opt their child out of the study and informed pupil assent was obtained.

### 2.2 Procedure

Participants completed questionnaires and computerized tasks in a classroom setting supervised by research assistants. Measures were completed prior to (baseline) and after the intervention (follow-up; on average nine weeks later).

### 2.3 Outcomes

#### 2.3.1 Depressive symptoms

Participants completed the short Mood and Feelings Questionnaire (Angold et al., 1995) about symptoms during the past 2-weeks (*α*=.90 baseline; *α*=.91 follow-up). A score of 11 or above indicates symptomatology within the clinical range (Angold et al., 1995).

#### 2.3.2 Reward-processing

Participants completed a shortened version of the Cambridge Gambling Task (CGT) www.camcog.co.uk (Clark et al., 2008; Rawal et al., 2013b). At the start of each block, participants receive 100 points and try to maximize points by betting on gambles involving two possible outcomes (winning or losing). Participants were told “the idea of the task is to build up as many points as you can. Try not to let your score get as low as 1 point because then you will lose the game.” On each trial, 10 colored boxes (blue or red) of varying ratios (9:1, 8:2, 7:3, 6:4, 5:5) are presented on screen. The first phase is a decision-making phase where participants must decide under which color the computer has hidden a token. The second phase is a reward-seeking phase where participants must bet a proportion of their points on the chosen color. Possible bets are offered in sequence (5%, 25%, 50%, 75%, 95% of points) in 2.5 s increments and the participant must select the magnitude of their stake. The amount bet is then added to (if correct) or subtracted from (if incorrect) the total score. Reward-seeking is measured by the proportion of points gambled on trials where the more likely outcome is selected (i.e. the tendency to risk existing points to accumulate further reward). Analysis of betting behavior was limited to trials where the participant selected the more likely outcome to maintain independence of reward seeking and decision-making. Trials where the ratio of boxes was equal (5:5) were excluded from analysis.

#### 2.3.3 Negative self-beliefs

The Dysfunctional Attitudes Scale for Children (DASC) (D’Alessandro and Burton, 2006) consists of 22 items (e.g. “I can be happy only if everybody I know likes me”) rated on a six-point scale (strongly disagree to strongly agree). Participants completed the DASC on touch-screen laptops to assess reaction time (Rawal et al., 2013a). The extent of agreement with each item was recorded in the usual questionnaire format as well as the response latency in milliseconds. Outcomes were: 1) total self-reported score (possible range 22–132 with higher scores representing more dysfunctional beliefs (*α*=.91 baseline; *α*=.92 follow-up). 2) The difference between mean latency to disagree with versus agree with negative self-beliefs. Larger relative reaction times indicate more reflective processing and are inversely related to adolescent depression (Rawal et al., 2013a; Sheppard and Teasdale, 2004).

#### 2.3.4 Over-general autobiographical memory

The “Sentence Completion for Events From the Past Test; SCEPT” was used as this is sensitive enough to detect over-general memory in non-clinical populations (Raes et al., 2007). 11 sentence stems probe past experiences (e.g. “I can still picture how….”; “I will never forget…”). Participants were instructed to complete the sentences and refer to a different topic for each. The outcome measure was the proportion of over-general memories (i.e. memories for repeated activities (e.g. “when I walk my dog”) and/or time periods longer than a day (e.g. “when I was on holiday last year”)). Coding was carried out as recommended (Williams et al., 2007) where responses referring to a particular time and place were coded as specific, responses that were categoric (referring to repeated events) or extended (referring to events occurring over long periods) were coded as over-general and responses that did not describe a memory were coded as semantic associates. 29 ratings were rated independently by two raters. Inter-rater agreement for the outcome measure of over-general versus specific was excellent =92%.

#### 2.3.5 Statistical analysis

We initially examined cross-sectional associations between cognitive variables and depressive symptoms at baseline. We next checked baseline condition differences on sociodemographic variables, cognitive risk factors and depressive symptoms. Depressive symptoms were skewed and square-root transformed for analyses. Descriptive statistics in tables are untransformed. Next, analyses examined whether cognitive variables changed following participation in each condition relative to the comparison condition (PHSE). To account for the hierarchical nature of the data (interventions conducted in multiple classes in each school), we used a series of linear random effects regression models. All analyses that follow include classroom as a random effect with the exception of models that focus on reward-seeking where reward ratio (9:1, 8:2, 7:3, 6:4) was nested within individuals and therefore individual was included as the random effect. Condition was dummy-coded with the PHSE comparison group as the reference group. Each model included age and gender as covariates in addition to relevant task-specific control variables as outlined in table legends. For the analysis of reward-seeking, interactions between condition type and reward-seeking ratio, modeled as a linear effect were included. This was done because previous research shows task familiarity (without any intervention) is related to increased reward-seeking at the most probable ratio of 9:1 (Rawal et al., 2014). Next we tested whether condition was associated with depressive symptoms at follow-up adjusting for depressive symptoms at baseline, age and gender. We also tested whether cognitive variables at baseline moderated depressive symptom change by including an interaction term between the baseline cognitive variable and condition in the regression models as described above. Finally, we tested whether change in the cognitive variables was associated with change in depressive symptoms in the hypothesized intervention groups. Cognitive change was calculated by subtracting baseline scores from follow-up scores (higher scores therefore represent greater change in the expected direction i.e. greater reward-seeking). Where there was evidence that cognitive change was associated with depression symptom change, we ran a structural equation moderated-mediation model to estimate the magnitude of the indirect path on depressive symptoms (via cognitive change) and if this differed by intervention group. Analyses were carried out in STATA version 13.

## 3 Results

256 adolescents participated (50 TRY; 53 CBT; 54 MBCT; 99 PHSE comparison). 236 (92%) and 227 (89%) completed pre- and post-intervention assessments and completion rates did not differ by group (Kendall's tau *c*=−.02, *p*=.53; −.04, *p*=.36 respectively).

Adolescents with higher depressive symptoms had more negative self-beliefs (*r*=.29, *p*<.001) and were quicker to agree than disagree with negative self-beliefs (*r*=−.34, *p*<.001) indicating an association between depressive symptoms and less reflective negative self-beliefs. Depressive symptoms were associated with lower reward-seeking at all probability ratios except the most uncertain (*r* (9:1)=−.15, *p*=.03; *r* (8:2)=−.15, *p*=.03; *r* (7:3)=−.20, *p*=.004; *r* (6:4)=−.12, *p*=.09). OGM was not associated with depressive symptoms (*r*=.03, *p*=.68).

Table 1 shows baseline group differences on demographic and cognitive variables and depressive symptoms. Groups did not differ in gender or household composition, but did differ in age. There were no group differences in depressive symptoms at baseline (*F*(3, 224)=.65, *p*=.58) or in the proportion above a clinical cut-point for depression (*χ*^2^(3)=.47, *p*=.93). There were no gender differences in total dysfunctional attitudes score, relative time to agree versus disagree, or over-general memory although reward-seeking was higher in boys and depressive symptoms were higher in girls. Gender and age were included as control variables in analyses.

### 3.1 Cognitive change following intervention

**Fig. 1:**
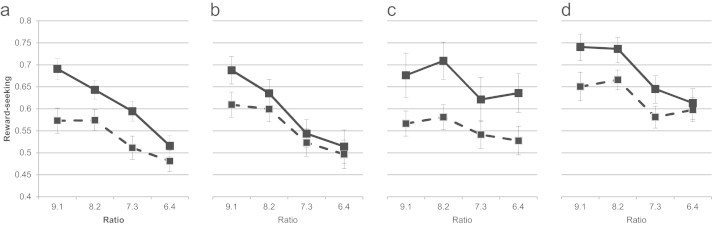
Reward-seeking pre- and post-intervention by group. Dashed line=reward seeking at baseline. Solid line=reward seeking at follow-up. a) Comparison; b) CBT; c) TRY; d) MBCT.

1. Reward processing
  Fig. 1 illustrates reward-seeking behavior by group at baseline and follow-up. Consistent with previous research (Rawal et al., 2014), increases in reward-seeking were greater at higher ratios regardless of group (*B*=.04, SE=.004, *p*<.001). TRY was associated with a significant increase in reward-seeking (*B*=.12, SE=.04, *p*=.01). Reward seeking did not change following CBT (*B*=−.05, SE=.04, *p*=.16) or MBCT (*B*=.04, SE=.04, *p*=.32). There was a significant interaction between group (TRY versus comparison) and ratio (*B*=−.03, SE=.01, *p*=.03). Follow-up analyses indicated that for TRY, ratio was not associated with increase in reward-seeking (*B*=.02, SE=.01, *p*=.17) whereas for the comparison group it was (*B*=.04, SE=.01, *p*<.001). Thus, unlike in other conditions, reward seeking increased in TRY regardless of the probability of reward.
2. Negative self-beliefs
  CBT showed non-significant lengthened reaction times to agree with versus disagree with negative self-beliefs at follow-up (CBT *B*=1081.88, SE=895.24, *p*=.23; Table 2).
3. Over-general autobiographical memories
  TRY and MBCT showed non-significant decreases in over-general autobiographic memory compared to comparison (TRY *B*=−.04, SE=.05, *p*=.45; MBCT *B*=−.03, SE=.05, *p*=.62; Table 2).

### 3.2 Change in depressive symptoms following intervention

Depressive symptoms changed differentially according to condition (*χ*^2^(3)=13.53, *p*=.004; Table 3). Compared to the comparison group, TRY led to a trend-level reduction in depressive symptoms at follow-up (*B*=−.36, SE=.20, *p*=.07) whereas, depressive symptoms showed an increase in MBCT (*B*=.44, SE=.19, *p*=.02) and no change in CBT (*B*=.19, SE=.19, *p*=.32). The importance of comparing interventions has been highlighted and therefore we compared change in depressive symptoms following each active intervention. TRY was associated with a significant reduction in symptoms compared to CBT (*B*=−.55, SE=.23, *p*=.02) and MBCT (*B*=−.80, SE=.23, *p*<.001). CBT and MBCT did not differ (*B*=−.25, SE=.22, *p*=.25).

### 3.3 Cognitive variables as moderators of depressive symptom change

Next, we carried out exploratory analyses to examine whether individual variation in cognitive variables at baseline moderated the observed changes in depressive symptoms for any of the intervention groups. Reward-seeking at baseline moderated depressive symptom change in the TRY and CBT groups (interaction terms TRY *B*=1.62, SE=.47, *p*=.001; CBT *B*=2.00, SE=.43, *p*<.001) but not in the MBCT group (interaction term *B*=−.42, SE=.48, *p*=.38). CBT and TRY were associated with a greater decline in depressive symptoms for pupils with lower reward seeking at baseline. Negative self-beliefs did not moderate change in depressive symptoms in any of the three intervention groups (interaction terms: CBT *B*=−.00001, SE=.0001, *p*=.92; TRY *B*=.0001, SE=.0001, *p*=.54; MBCT *B*=−.00001, SE=.0001, *p*=.94). Over general autobiographical memory did not moderate any of the three intervention groups (interaction terms: CBT *B*=.58, SE=1.15, *p*=.61; TRY *B*=−.81, SE=.88, *p*=.36; MBCT *B*=−.54, SE=.95, *p*=.57).

### 3.4 Cognitive change and change in depression

We next tested our hypothesis that change in reward-seeking would be associated with change in depression in the TRY group. Change in reward-seeking was associated with change in depression (*B*=.92, SE=.24, *p*<.001; Table 4) and this relationship differed significantly between TRY and comparison (*B*=−2.42, SE=.43, *p*<.001). Follow-up analyses indicated that for TRY, increased reward-seeking was associated with decreased depressive symptoms (*B*=−1.41, SE=.41, *p*=.001). Finally, we ran a mediation model in which change in reward-seeking mediated change in depressive symptoms, to test whether TRY moderated the pathway from change in reward seeking to follow-up depressive symptoms. There was significant moderation (*B*=−2.22, SE=.65, *p*=.001) where the magnitude of the indirect effect via change in reward-processing was stronger in TRY (*B*=−.02, SE=.01, *p*=.12) than in all other groups (*B*=−.0003, SE=.01, *p*=.96).

Change in the other two cognitive variables was not associated with depressive symptom change (results available from first author).

## 4 Discussion

There are considerable benefits to understanding the processes that underlie symptomatic change in interventions because this facilitates intervention innovation (Hollon et al., 2002; Kraemer et al., 2002; Rutter, 2007). We sought to identify which cognitive risk-factors were changed by different types of universal preventive intervention (TRY, CBT and MBCT). We predicted that the three interventions would differentially alter reward-seeking, negative self-beliefs and over-general autobiographical memory respectively given their different content and focus. Results suggested that incorporating reward-processing into preventive interventions for adolescent depression could improve efficacy. First, only the intervention that explicitly focused on enhancing sensitivity to reward (TRY) was associated with a post-intervention decrease in depressive symptoms. Although this reduction was not significantly greater compared to comparison (*p*=.07) it was superior to that observed for the other prevention programs (*p*<.05). Second, reward-seeking behavior increased following participation in TRY. Degree of change in reward-seeking was associated with improvement in depressive symptoms and this association differed significantly for TRY and comparison. Reward-seeking at baseline was also the only cognitive variable to moderate depressive symptom change. Taken together, these results suggest that reward-seeking may underlie symptom change in the TRY intervention.

Considering what did not change following intervention is useful for informing what may not work in unselected universal interventions. Evidence for negative self-beliefs and over-general autobiographical memory as mechanisms underlying symptomatic change in universal prevention programs was equivocal. Our results are consistent with evidence from large trials indicating that CBT may not be effective in *unselected* groups of adolescents (Stallard et al., 2012). We hypothesized that negative self-beliefs would become more reflective following CBT given the focus on activities encouraging the identification and evaluation of negative self-beliefs and cognitive distortions. Greater reflective processing of negative emotional material may contribute to effective emotion regulation (Sheppard and Teasdale, 2004; Teasdale, 1999). We observed a non-significant slowing of the time taken to endorse negative attitudes in this group and depressive symptoms were not changed following CBT. Similarly, over-general autobiographical memory did not significantly reduce following MBCT. Although MBCT has been shown to be efficacious in preventing relapse in adults with a history of recurrent depression (Piet and Hougaard, 2011; Williams and Kuyken, 2012), it not been widely used as a prevention method in adolescents. One recent study reported promising results for a mindfulness intervention delivered in schools expressing an interest in mindfulness (Kuyken et al., 2013) although we did not select schools in this way. In fact, we observed a post-intervention increase in depressive symptoms following MBCT. This was unexpected and as we did not follow-up individuals postintervention, it is not clear whether this would persist over time. It is also possible that an increase in depressive symptoms following MBCT, which focused on increasing awareness and acceptance of feelings through meditation, could reflect greater recognition of and attentiveness to existing symptoms of depression. Although our results require replication, they are consistent with ideas that mindfulness and meditation may not be beneficial for all (Farias and Wikholm, 2015). Indeed, a recent trial in adults showed that MBCT was only effective at preventing depression relapse in particularly vulnerable individuals (those with a history of childhood trauma) (Williams et al., 2014). Relatedly, an additional explanation for the lack of change in OGM following MCBT concerns recent findings suggesting that OGM may act as a risk factor for depression only in certain high-risk groups of youth and not in general community samples (Crane et al., 2015). This is consistent with results of the current study where OGM was the only potential cognitive risk factor examined that showed a non-significant association with depressive symptoms at baseline.

Collectively, results suggest that incorporating reward-related activities into prevention programs may be a way of enhancing efficacy when interventions are delivered to unselected populations of adolescents. The TRY intervention aimed to incorporate elements of rational reward-seeking behavior (i.e. encouraging young people to consider the likelihood of good or bad outcomes in their reward-seeking behavior) based on evidence that low reward-seeking may be a causal risk factor for adolescent depression (Forbes et al., 2007; Rawal et al., 2013b). It is possible that some programs, such as those that focus on challenging negative thoughts, may be effective prevention methods for adolescents with sub-clinical symptoms but not for unselected populations where the majority of individuals will not be experiencing overt symptoms.

This study has a number of strengths. First, it incorporates state-of-the-art behavioral assessments of putative causal mechanisms into a longitudinal design. Such a design allows the elements altered by an intervention to be elucidated and is an essential step in understanding how interventions produce effects on symptoms (Kraemer et al., 2002). Behavioral measures may also be more sensitive at detecting cognitive change than self-report measures (Harmer et al., 2009; Rawal et al., 2013a). Second, it directly compares a number of differing preventive interventions which allows comparison of the variables that they change as well as their effect on depressive symptoms while controlling for non-specific intervention effects (Merry and Stasiak, 2012; Stallard et al., 2012). We required *written* pupil assent but did not require *written* parental consent in order for adolescents to participate in the study. This has the advantage of facilitating pupil participation from a range of demographic backgrounds – but whether this approach can be used will depend on local ethical regulations. Nonetheless, a number of limitations should be acknowledged. Sample sizes in the different intervention groups were relatively small which may have limited power to detect effects. The study used a non-randomized design which limits the ability to make causal inferences. The major issue with non-randomized studies is the possibility of differences on key confounders across groups. However, the obvious potential confounder in this study was depressive symptoms at baseline which were controlled for in analyses and for which there were no pre-intervention group differences. In fact, there were no significant pre-intervention group differences on key study variables except OGM.

## 5 Conclusion

Effective programs to prevent depression in teenagers would reach large numbers of individuals if delivered as part of the school curriculum. This study showed preliminary evidence to suggest that reward-sensitivity can be altered by intervention and the degree of change is associated with depressive symptom change. Further research is required to evaluate whether incorporating training in reward-processing is a promising avenue for consideration in universal prevention trials for adolescent depression.

## Role of funding

This work was funded by an MRC Centenary Award to FR (G0802200). The funder had no role in the design and conduct of the study; collection, management, analysis, and interpretation of the data; and preparation, review, or approval of the manuscript

## Conflicts of interest

The authors have no conflicts of interest.

## Contributors

FR obtained funding. FR and SD designed the study. AR collected data. LR, GL and FR performed statistical analyses. Glyn Lewis provided statistical advice. All authors contributed to writing the manuscript and have approved the final version. FR had full access to all of the data in the study and takes responsibility for the integrity of the data and the accuracy of the data analysis. All authors report no conflicts of interest.

## Figures and Tables

**Table 1:** Demographics, cognitive variables and depressive symptoms at baseline by group (mean (SD)).

	Comparison	CBT	TRY	MBCT	
*Age* (%)					*χ*^2^(3)=16.78, *p*=.001
13	42	37	24	17	
14	40	12	19	32	
*Gender* (%)					*χ*^2^(3)=1.90, *p*=.59
Male	45	30	21	26	
Female	51	23	26	24	
*Household* (%)					χ^2^(3)=.64, *p*=.89
Mother and father	54	29	23	28	
Single parent/other	35	21	20	21	
*Reward seeking by ratio of reward*					
*Reward 9:1*	.58 (.25)	.62 (.19)	.58 (.23)	.64 (.22)	*F*(3, 225)=1.01, *p*=.39
*Reward 8:2*	.59 (.22)	.63 (.20)	.59 (.19)	.65 (.16)	*F*(3, 225)=1.06, *p*=.36
*Reward 7:3*	.54 (.24)	.54 (.21)	.54 (.20)	.58 (.17)	*F*(3, 225)=.36, *p*=.78
*Reward 6:4*	.52 (.23)	.50 (.22)	.53 (.20)	.59 (.18)	*F*(3, 225)=1.78, *p*=.15
*Negative self-beliefs*					
(reaction times)	937.14 (1841.06)	863.94 (1831.87)	914.16 (2191.44)	1196.49 (2214.48)	*F*(3, 157)=.13, *p*=.94
*OGM*	.35 (.23)	.35 (.21)	.49 (.25)	.40 (.22)	*F*(3, 179)=3.45, *p*=.02
*Depression*	6.02 (5.95)	5.40 (5.57)	5.88 (5.36)	4.98 (5.42)	*F*(3, 223)=.65, *p*=.58
*% clinical cut point*	17	20	19	15	*χ*^2^(3)=.47, *p*=.93

OGM=over general memory; Depression=depressive symptom score on short Mood and Feelings Questionnaire; % depressed=proportion scoring at or above the clinical cut-point of 11 on the short Mood and Feelings Questionnaire.

**Table 2:** Change in cognitive variables.

	Mean at follow-up (SD)				Regression results		
*B* (SE)	*p*	95% CI
***Reward-seeking***	9:1	8:2	7:3	6:4			
*Model* 1							
*Comparison*	.68 (.21)	.64 (.19)	.58 (.20)	.54 (.21)	−.05 (.04)	.16	−.13, .02
*CBT*	.69 (.21)	.64 (.21)	.55 (.21)	.52 (.26)
*TRY*	.68 (.28)	.70 (.24)	.62 (.26)	.62 (.24)	.12 (.04)	.01	.03, .20
*MBCT*	.72 (.21)	.73 (.17)	.65 (.20)	.61 (.21)	.04 (.04)	.32	−.04, .11
*Ratio*					.04 (.01)	<.001	.03, .05
CBT*Ratio					.01 (.01)	.44	−.01, .03
TRY*Ratio					−.03 (.01)	.03	−.05, −.002
MBCT*Ratio					−.04 (.01)	.71	−.02, .02
***Negative self-beliefs*****(*****RT*****)**							
*Model* 2							
Comparison		1117.76 (2388.44)					
CBT		2678.25 (5688.63)		1081.88 (895.24)	.23	−672.77, 2836.52	
TRY		1134.32 (295.00)		−768.00 (907.70)	.40	−2547.06, 1011.07	
MBCT		1317.54 (2688.39)		−718.55 (1102.69)	.52	−2879.78, 1442.68	
***Over-general memory***							
*Model* 3							
Comparison		.39 (.25)					
CBT		.32 (.26)			.01 (.06)	.85	−.10, .12
TRY		.43 (.26)			−.04 (.05)	.45	−.15, .07
MBCT		.32 (.24)			−.03 (.05)	.62	−.13, .07

Model 1 adjusts for gender; age, baseline reward seeking, and quality of decision making (i.e. the % of trials on which the more likely of the two colors (blue or red) was chosen). Negative self-beliefs RT=reaction time to agree versus disagree with dysfunctional attitudes where larger reaction times index more reflective processing. Model 2 adjusts for age, gender, baseline total dysfunctional attitudes score, baseline number of agreements on dysfunctional attitudes scale and baseline reaction time different to agree with versus disagree with dysfunctional attitudes. Model 3 adjusts for age, gender and baseline over-general memory.

**Table 3:** Depressive symptoms at baseline and follow-up (mean (SD)).

	Comparison	CBT	TRY	MBCT
*Depressive symptoms*				
Baseline	6.02 (5.95)	5.40 (5.57)	5.88 (5.36)	4.98 (5.42)
Follow-up	4.93 (4.77)	5.29 (5.72)	3.79 (5.09)	6.46 (6.09)
% *clinical cut point*				
Baseline	17	20	19	15
Follow-up	13	16	5	20

% clinical cut point=proportion scoring at or above the clinical cut-point of 11 on the short Mood and Feelings Questionnaire.

**Table 4:** Change in cognitive risk factors predicting change in depressive symptoms.

***Reward seeking***	*B* (SE)	*p*	95% CI
*Model* 1			
Change in reward-seeking	.92 (.24)	<.001	.45, 1.40
CBT	.21 (.10)	.03	.02, .39
TRY	−.14 (.11)	.20	−.36, .08
MBCT	.45 (.10)	<.001	.21, .64
CBT*change in reward-seeking	−.92 (.47)	.05	−1.84, .01
TRY*change in reward-seeking	−2.42 (.43)	<.001	−3.27, −1.57
MBCT*change in reward-seeking	−.22 (.45)	.62	−1.10, .65

Model 1 adjusts for gender; age, the ratio of reward (i.e. 9:1, 8:2, 7:3, 6:4, recoded as 4, 3, 2, 1 respectively) baseline depressive symptoms and quality of decision making (i.e. the % of trials on which the more likely of the two colors (blue or red) was chosen).
