# Supplementary material for: Examining reward-seeking, negative self-beliefs and over-general autobiographical memory as mechanisms of change in classroom prevention programs for adolescent depression
Source: J Affect Disord. 2015 Nov 1;186:320–7. doi: 10.1016/j.jad.2015.07.019 (PMC4573464; doi:10.1016/j.jad.2015.07.019)
Supplement: Supplementary file 1 — Supplementary material [file mmc1.doc]

**Appendix 1**

**Intervention material development**

In developing the manuals and materials for use in the present study, we formed a panel of researchers and therapists with expertise in this area (FR, AR, SD, AC, JM). We carried out a literature review of prevention trials for adolescent depression. We identified features that characterised good therapeutic practice and factors associated with intervention outcome through literature review and expert knowledge. We used knowledge about the aetiology of adolescent depression to evaluate the inclusion of particular activities. We carefully examined programs that had previously been shown to be effective at preventing adolescent depression in at least two independent studies. There were two such programs and both were CBT programs.

The present study was designed to compare standard CBT, CBT with a focus on behavioural activation and reward decision-making and mindfulness-based CBT. All programs included eight sessions. Manuals for group leaders and workbooks for adolescents were developed for all interventions. In each intervention the first session involved psychoeducation about adolescent depression and included ‘ice-breaker’ exercises for the group to get to know each other and the therapist. Following this, the CBT intervention, proceeded along the following lines: sessions 2-5 involved introducing and extending the CBT model (i.e. the relationship between body signals, thoughts, feelings and behaviour), the role of stressful events play in this model and identifying, evaluating and challenging negative thoughts. Session 6 involved practicing these skills in finding solutions to problems. Session 7 involved identifying and using social support. Session 8 involved a recap of material. The CBT intervention therefore primarily focused on identifying, evaluating and challenging negative thoughts with an additional session about social support.

TRY involved: introducing a modified CBT model (i.e. the relationship between body signals, thoughts, feelings and *decisions*); a session on identifying rewarding experiences and happy memories; a session on identifying and evaluating thoughts; two sessions on decision making (evaluating potential risks and rewards when making decisions); a session on the role of social support and a final recap session. Three sessions were the same as the standard CBT manual (session 1, psychoeduation; session 4, negative thoughts; and session 7, support networks). TRY primarily involved identifying and focusing on positive events and memories in addition to decision making training. Additional information including content such as video clips used in the intervention is available at <http://www.ucl.ac.uk/educational-psychology/try.html>.

MCBT involved: a session introducing the idea of being mindful and paying attention to body sensations focusing on the breath; a session about keeping calm (using the breath) and stress triggers; a session on switching attention; a session introducing the CBT model; two sessions on mindfulness of thoughts and feelings and a final recap session. MCBT primarily involved increasing awareness and acceptance of bodily sensations, thoughts and feelings.

**Therapist training and supervision**

**Therapist training and supervision**

All therapists were qualified educational psychologists working in local authority schools with additional training in CBT. All therapists attended a full day training session prior to the start of the intervention delivery where they were introduced to the theory and research underpinning it and became familiar with the programme materials. All therapists attended group supervision meetings with an experienced CBT trainer and practitioner and UK Health and Care Professions Council (HCPC) registered Psychologist (SD). Two hour group supervision sessions took place three times during the 8 week intervention (after sessions 2, 4 and 6). The aim of these sessions was to ensure consistency of application of the principles and practice of CBT, and to learn from others’ experiences of delivering the intervention, managing group dynamics and school personnel. Within the group supervision, agendas were set at the start and experiences shared. The supervisor facilitated the discussion and aimed to clarify and ensure consistency by eliciting examples of best practice from group members, attending to the experience of delivering the intervention and maintaining records of the key areas discussed.

**Fidelity of intervention delivery**

An early and a late session from each intervention was videotaped. An independent rater not involved in the intervention rated manual adherence, facilitator and adolescent engagement for each session. Descriptors e.g. “Adolescents are engaged and compliant. They appear to complete tasks set effectively” were rated on a four point scale ranging from 1 (weak evidence) to 4 (strong evidence). These were converted into percentage scores by dividing mean scores by the total possible score. Adherence to manual content was assessed by three items: clear instructions and aims, effective use of program materials and intervention objectives met (α = .78). Adolescent engagement was assessed with the following items: engagement, contributions from a range of individuals and paying attention (α = .90). Facilitator engagement was assessed with the following items: enthusiasm, engagement and acknowledgement of verbal contributions (α = .65). A proportion of tapes were independently rated by a second rater and the intra-class correlation (ICC) assessed inter-rater reliability for each scale (ICC=.706; .906; .789). Mean scores did not differ by intervention condition (F=.713, p=.519 adherence; F=.576, p=.584 adolescent engagement; F=.315, p=.738 facilitator engagement).
